# DNA sensors, crucial receptors to resist pathogens, are deregulated in colorectal cancer and associated with initiation and progression of the disease

**DOI:** 10.7150/jca.34188

**Published:** 2020-01-01

**Authors:** Liangmei He, Yuxia Liu, Weiling Lai, Hongbo Tian, Lingxia Chen, Lu Xie, Zhiping Liu

**Affiliations:** 1 Department of Gastroenterology, The First Affiliated Hospital of Gannan Medical University;; 2 Center for Immunology, Key Laboratory of Prevention and Treatment of Cardiovascular and Cerebrovascular Diseases, Ministry of Education, Gannan Medical University; 3 Gannan Medical University;; 4 School of Basic Medicine, Gannan Medical University, Ganzhou, Jiangxi, 341000 China.

**Keywords:** DNA sensors, expression profile, cancer, cancer stages

## Abstract

**Background:** DNA sensors are innate immune receptors that detect intracellular endogenous or exogenous DNA. They are critical to trigger immune response against DNA viral and intracellular bacterial infection, and are involved in inflammatory diseases and tumorigenesis. Recent accumulating evidences indicated that DNA sensors are also crucial for controlling the development of colorectal cancer (CRC). However, a systematic study on the expression profile of DNA sensors in CRC and their clinical significance are still lacking.

**Methods:** We investigated the expression profile of DNA sensors in CRC and their clinical significance by taking advantage of clinical CRC samples, mouse AOM/DSS treatment model, and Oncomine ® bioinformatics platform.

**Results:** Our study identified that the expression of DNA sensors, including *AIM2, DAI,* as well as inflammasome molecules *ASC/IL-18*, *TLR9* and adaptor *MyD88*, and *DDX60* decreased in human CRC, whereas the expression of *DHX9*, *DHX36*, and *DDX41* significantly increased. Among them, the expression of *AIM2/ASC/IL-18*, *MyD88*, *DAI*, *DHX36*, and *DDX60* were associated with cancer stages. In addition, we also performed correlation analysis between DNA sensors and their main signaling molecules to explore the possible mechanisms. The results showed that there were positive correlations between *AIM2* and *ASC/IL-18*, *DHX9* and *MAVS*, and *TLR9* and *MyD88* expression. In addition, the gene expression patterns of some DNA sensors were confirmed by Western-blot analysis.

**Conclusions:** Our study revealed that the expression of multiple DNA sensors was deregulated in CRC and might be involved in tumor development. More importantly, the study identified that, among all these DNA sensors, AIM2, DAI, and DDX60 could be potentially critical for diagnosis, prognosis, and therapy of CRC and deserve further investigation.

## Introduction

Colorectal cancer (CRC) is one of the major diseases that are associated with cancer-related death throughout the world. Over 1.8 million new colorectal cancer cases and 881,000 deaths are estimated to occur in 2018 1. Although the incidence and mortality of CRC have declined in some developed countries in recent years, they still show an upward trend in some developing or under-developed countries 2. When most of the patients are diagnosed with CRC, they have been already in the advanced stage. Therefore, the combination of surgery treatment and postoperative chemotherapy are often required and the treatment effect is usually not optimal. All these factors have brought a heavy burden to the society and economic health. Therefore, exploring new molecular mechanisms of CRC development may provide new ideas and targets for the diagnosis and treatment of CRC.

The onset of CRC is a multistep, multifactorial, and polygenic process, but chronic inflammation is well recognized as a risk factor for CRC. Clinically, inflammatory bowel disease (IBD) patients are more likely to develop into CRC than healthy people 3. The IBD mouse model also supports the notion that inflammation can promote the development of CRC 4. The innate immune receptors, pattern recognition receptors (PRRs), are the key factors to trigger the inflammatory response 5. In addition to regulating inflammation development, some PRRs also play an important role in the process of cell proliferation and apoptosis 6. These receptors mainly include Toll like receptors (TLRs), Nod-like receptor (NLRs), RIG-I like receptor (RLRs), AIM2 like receptor (ALRs), and C-type lectin receptor (CLRs). After activation by the pathogen-associated molecular patterns (PAMPs) or danger-associated molecular patterns (DAMPs), PRRs can induce self-activation and cytokine release, and further activate the adaptive immune cells, so as to mount potent antiviral immune response 7 .

The DNA sensors are one of the major types of innate immune receptors. Most of the DNA sensors are present and functional in the cytoplasm 8, 9. DNA sensors mainly include ALRs, such as Absent in melanoma 2 (AIM2) and Interferon gamma inducible protein 16 (IFI16), cGAMP synthase (cGAS), Z-DNA binding protein 1(ZBP-1 or DAI), Toll like receptor 9 (TLR9) and helicase family members, such as DEAH-Box Helicase 9 (DHX9), DEAD-box helicase 41(DDX41), and DEAD-box helicase 60 (DDX60) 10. They can detect abnormal intracellular endogenous or exogenous nucleic acids, and then induce activation of the inflammasome or the production of type I interferons (type I IFNs) and other inflammatory cytokine through a series of downstream adaptor molecules, thus effectively defensing host against intracellular bacterial and viral infection 11.

However, recent accumulating evidences showed that DNA sensors, such as AIM2, TLR9, and adaptor molecule STING, are important not only for host defense against pathogens, but also for controlling the development of colitis and CRC 9, 12, 13, 14.

AIM2 recognizes double-stranded DNA (dsDNA) to mediate the assemble of a multi-protein complex, inflammasome, through binding to apoptosis-associated speck-like protein containing a CARD (ASC) and Caspase-1, which then cleaves pro-IL-1β and pro-IL-18 into mature forms 15. However, studies have shown that AIM2 inhibits the occurrence of CRC by regulating intestinal epithelial cells via an inflammasome-independent manner 16, 17, 18. STING is a cytoplasmic receptor for cyclic dinucleotides and an important adaptor protein of other DNA receptors, such as DAI, cGAS, DDX41, and IFI16 19, 20, 21, 22. These upstream DNA receptors transmit signals through STING and modulate the production of type I IFNs. STING inhibits the development of CRC by inhibiting intestinal inflammation and stimulating the production of type I IFNs 23, 24, 25.

In addition, studies have shown other DNA sensors, such as DHX9, DDX41, and DDX60, also play important roles in the development of tumors 26, 27, 28. Therefore, we hypothesize that DNA sensors may play important roles in the development of colitis and CRC. However, in general, most of the signaling pathways of these DNA sensors and their roles in the development of CRC remain largely unknown. Furthermore, whether these DNA sensors in CRC tissues and normal tissues are differentially expressed, and whether their expressions are associated with clinical stages, grades, and prognosis, are yet to be investigated.

In this study, the expression AIM2, IFI16, DAI, TLR9, cGAS, DHX9, DHX36, DDX41, DDX60 of DNA sensors as well as inflammasome molecules ASC/IL-18, TLR9 and adaptor MyD88, and adaptor molecule STING in human were examined. We also took the advantage of Oncomine data platform to further analyze their correlation with clinical cancer stages. Oncomine is a large bioinformatics integration platform, which is designed to collect, standardize, analyze and communicate transcription data to biomedical researchers 29. Finally, we used early-stage mouse CRC models to investigate whether DNA sensors are involved in the formation of CRC. This study can not only benefit us for better understanding on the expression profile of DNA sensors in CRC, but also provide a theoretical basis for the diagnosis, prognosis, and treatment of CRC.

## Materials and Methods

### Clinical sample collection

CRC tissues and adjacent tissues were collected from Department of Gastrointestinal Surgery in the First-Affiliated Hospital of Gannan Medical University (Ganzhou, Jiangxi, China) (**Supplemental Table 1**). Patients who were newly diagnosed with CRC based on the clinicopathological criteria were included. Patients who received preoperative chemo-, radio- or immunotherapy were excluded. Upon resected, the cancer and the adjacent normal tissues (above 3cm away from cancer tissue) were stored in liquid nitrogen for subsequent qRT-PCR analysis.

### AOM (Azoxymethane) /DSS (Dextran sodium sulfate) treatment

AOM and DSS treatment by one dose of carcinogen AOM injection and three rounds of DSS treatment in drinking water is a well-established colitis-associated CRC animal model 30. In order to study the causes, not the results, of CRC development, AOM and DSS treatment by one dose of AOM injection and one round of DSS treatment (14 days after AOM treatment) is often used to study pre-cancerous mucosal gene expression changes 17, 31-33.

The 6-8 weeks old C57 BL/6 mice were divided into the experimental group and the control group. The experimental group received intraperitoneal injection of AOM (Sigma, USA, 10ug/g body weight), and the control group was injected with PBS. Five days after AOM injection, the mice in experimental group were fed up with 3% DSS water (MP Biologicals, USA), and the control group was fed up with regular drinking water. After 6 days, the 3% DSS water was replaced by regular drinking water for 3 days, and then some mice were sacrificed (day 14). The colorectal tissues were collected for further analysis.

### qRT-PCR

The total RNA of tissues was extracted using Trizol reagent (Invitrogen, USA). The cDNA was synthesized by M-MLV First Strand Kit (Invitrogen, USA). Then the Real-time PCR of DNA sensors were performed in Bio-rad Real-Time PCR System with the Sybergreen Master Mix (Invitrogen, USA). The primer sequences of DNA sensors were shown (**Supplemental Table 2, 3**). The β-actin was chosen as a reference gene. The relative expression of target genes in cancer and the control groups was expressed as 2^-ΔΔCT^.

### Data collection from Oncomine

Oncomine (https://www.oncomine.org) is the largest cancer microarray integrated platform worldwide, which aims to dig for cancer genetic information. So far, 729 gene expression datasets, more than 90000 cancer tissues and normal tissue samples were collected in this platform. In this study, the TCGA database in the Oncomine platform was chosen to study the expression profile of the DNA sensors (Analysis Type: Cancer vs. Normal Analysis; Cancer Type: Colorectal Cancer; Data Type: mRNA; Sample Type: Clinical Specimen; Genes: every one DNA sensors; Dataset name: TCGA Colorectal, Threshold fold change ≥ 1.5, p value ≥ IE-4, Gene Rank in the top 10).

### Western-blot analysis

Proteins were extracted from colorectal tissues by RIPA lysis buffer with proteinase inhibitors. Samples were resolved in SDS-PAGE and transferred onto NC membranes. Membranes were blocked in 5% milk for 1 hour and incubated in primary antibodies overnight at 4 degree. Membranes then were incubated with HRP-conjugated secondary antibody for 1 hour, and proteins were visualized using ECL substrates. The primary antibodies include STING (13647, Cell Signaling), IFI16 (ab55328, Abcam), DAI (sc-271483, Santa Cruz), DDX41 (15076, Cell Signaling), DHX9 (12266, Affinity), DHX36 (ab70269, Abcam), DDX60 (ab139807, Abcam) and β-ACTIN (A5441, Sigma).

### Data analysis

All data were analyzed in statistical software Prism 7.0. The quantitative data were represented by Mean ± SEM. As for qRT-PCR results, the relative expression of target genes in cancer and the control groups was expressed as 2^-ΔΔCT^. The expression differences of DNA sensors between human CRC tissues or AOM/DSS treated mouse tissues and the controls were analyzed by paired t test or unpaired t test, respectively. Quantitative data between groups were analyzed by ANOVA (variance). When the correlation between molecules was analyzed, the two sets of data were sequenced according to the order of the samples, and the correlation between the molecules was analyzed by multivariate linear regression analysis (Liner, regression). *p*<0.05 was considered statistically significant. *p < 0.05, **p < 0.01, ***p <0.001.

## Results

### The expression profile of AIM2 and inflammasome-associated molecules in CRC

**Figure 1:**
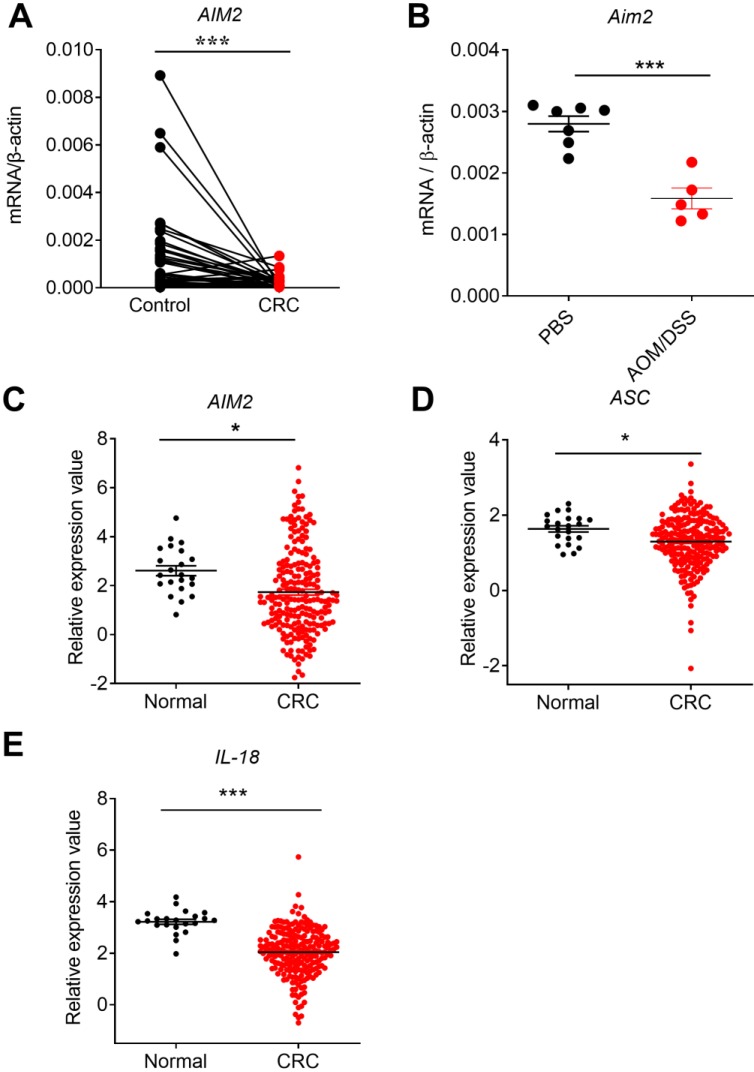
** The expression profile of *AIM2* in human colorectal cancer (CRC) tissues, mouse CRC tissues at early stage, and TCGA database.** RNA was extracted from cancer and matched peri-carcinomatous tissues of CRC patients, as well as tissues from AOM/DSS treated mice, and reverse-transcribed into cDNA. Then the gene expression levels of *AIM2* were determined by quantitative fluorescent PCR. The data from the same patient were connected by straight lines. The data of the expression of *AIM2* and inflammasome molecules *ASC* and *IL-18* were retrieved from the TCGA microarray database in Oncomine® platform. **(A)** *AIM2* expression in human CRC tissues, **(B)** *Aim2* expression in tissues from AOM/DSS treated mice, **(C)** *AIM2* expression in TCGA database, **(D-E)** The expression of inflammasome molecules *ASC* and *IL-18* in TCGA database. Control: matched peri-carcinomatous tissues, n=44 for clinical samples, data represent two independent experiments; n=7 for PBS-treated mice and n=5 for AOM/DSS- treated mice; n=22 for health normal controls and n=215 for CRC group. *p < 0.05, ***p < 0.001.

AIM2 is a cytoplasmic DNA sensor that mainly functions via inflammasome. An European study showed that low level of AIM2 in colon tissues of CRC patients was associated with poorer prognosis 34. However, genetic and epigenetic difference among regions and racial may exist 35. Recent study had shown that AIM2 and adjacent normal controls were not significantly different in Chinese CRC patients 36 . qRT-PCR results of our CRC samples showed that the expression of *AIM2* significantly decreased in CRC tissues, in comparison with the controls (**Figure 1A**). The sample content and quality difference may also contribute to this discrepancy. More studies are required to investigate the AIM2 expression in Chinese CRC patients.

**Figure 2:**
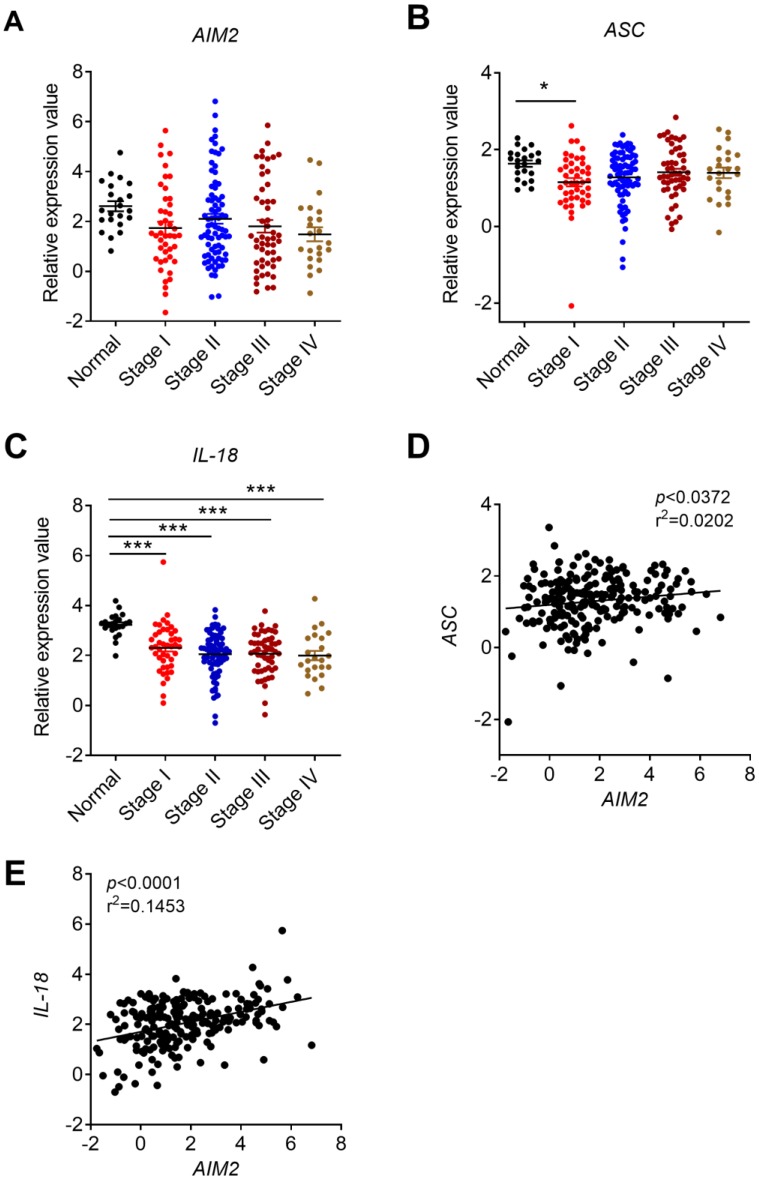
** Correlation analysis between the expression of AIM2 inflammasome molecules and CRC stages, and correlation between AIM2 and inflammasome molecules.** The expression data of *AIM2* and inflammasome molecules, *ASC* and *IL-18*, were retrieved from the TCGA microarray database in Oncomine® platform, and were grouped by stages or patient IDs for further analysis. **(A-C)** Correlation analysis between the expression of *AIM2*, inflammasome molecules (*ASC* and *IL-18*) and cancer stages were shown. **(D-E)** Correlation between the expression of *AIM2* and *ASC/IL-18*. n=22 for health normal control, 44 for stage I, 78 for stage II, 52 for stage III, and 23 for stage IV. Data were expressed as mean ± SEM, Log2 median-centered ratio expression. *p < 0.05; ***p < 0.001.

To further study whether the expression changes of *AIM2* in CRC were a universal phenomenon, we carried out a large sample analysis by taking advantage of Oncomine platform. Oncomine is the largest integration bioinformatics platform for microarrays, where the data were standardized uniformly 29. We chose the TCGA sub-database, including data from 215 CRC samples and 22 healthy samples, to further analyze the expression profile of DNA sensors. Consistently, we found that the expression of *AIM2* was significantly reduced in CRC in TCGA database (**Figure 1C**). Similarly, the expression of AIM2 inflammasome downstream molecules, *ASC* and *IL-18*, decreased in CRC (**Figure 1D**, **E**). Subsequently, we analyzed the expression of *AIM2*, *ASC*, and *IL-18* in different stages of CRC. However, in contrast to *ASC* and *IL-18*, the expression of *AIM2* in different stages of CRC was not significantly different from healthy control group (**Figure 2A, B, C**). *IL-18* displayed statistically different expression levels between healthy control and various CRC stages (**Figure 2C**), whereas only the *ASC* expression in CRC stage I was significantly different from health control (**Figure 2B**).

Previous *in vivo* studies found that the preventive effect of AIM2 in mouse CRC is independent of inflammasome activation and may be associated with the inhibition of AKT proliferation signaling pathway 16, 17. A recent *in vitro* study also found that up-regulation of AIM2 in CRC cell line HCT116 promoted cell apoptosis that may be associate with decreased AKT level 18. However, the formation and progression of tumor is determined by the complicated interaction between tumor and tumor microenvironments. Whether the preventive role of AIM2 in CRC is associated with inflammasome is still controversial. Our current results showed a positive correlation between *AIM2* and inflammasome-associated molecules *ASC* and *IL-18* in CRC (**Figure 2D, E**). These results indicate that AIM2 in CRC samples may function in either inflammation-dependent or inflammation-independent manners.

On the other hand, the gene expression change may reflect either the causes of tumor or the results of tumor formation. To further investigate whether AIM2 is involved in early stage of CRC formation, we employed the classical mouse inflammation-associated CRC model induced by AOM and DSS treatment. The mice were sacrificed at day 14 after a dose of AOM injection and one round of DSS treatment, which is a critical time point often used to study factors to induce the development of CRC 17, 31-33. The body weight loss and shortening of the colon are two of the major indexes of inflammation 30. The AOM/DSS-treated mice lost more body weight, displayed shorter colon lengths over the process of AOM/DSS treatment. In addition, H&E staining results showed that the normal colonic mucosa structure of the experimental group was disrupted 37. Subsequent qRT-PCR results showed that the colonic expression of *Aim2* was significantly downregulated in AOM/DSS-treated group in comparison with control groups (**Figure 1B**). In summary, our study showed that AIM2 and AIM2 inflammasome downstream molecules were downregulated in CRC samples, indicating that they may be involved in CRC formation and progression.

### The expression profile of STING signaling-associated molecules in CRC

**Figure 3:**
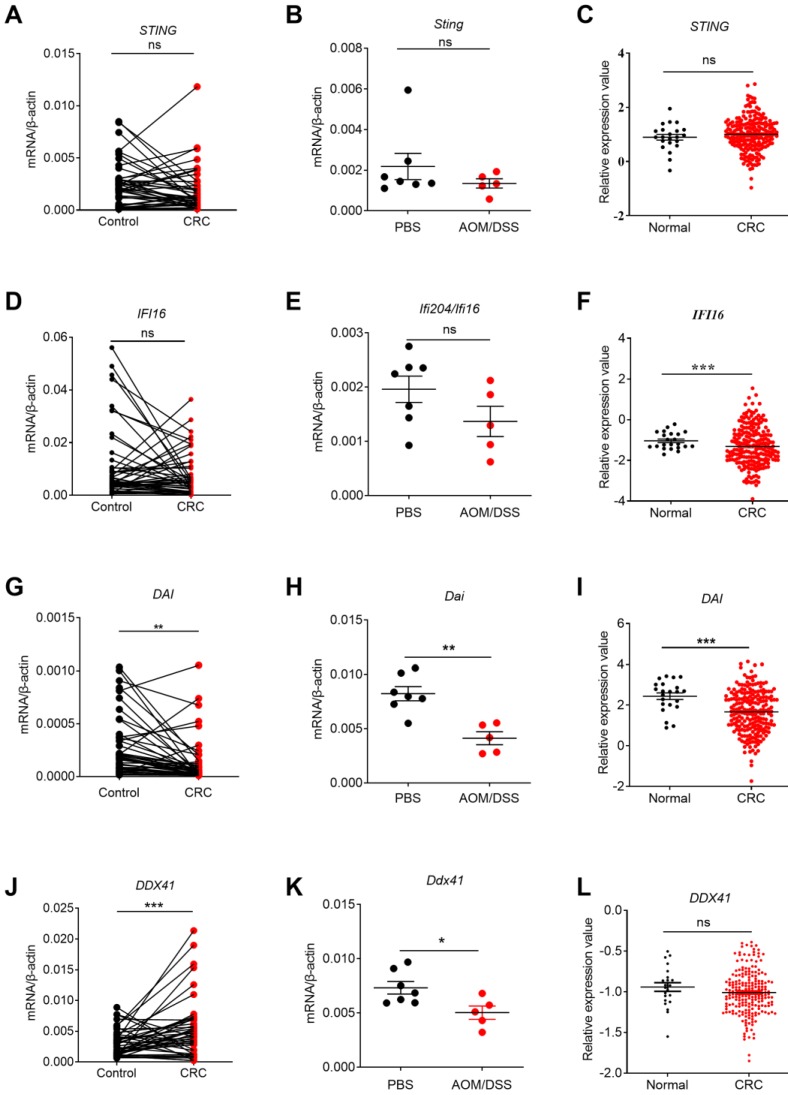
** The expression profile of STING signaling pathway-associated molecules in human and mouse CRC tissues and TCGA database.** RNA was extracted from cancer and matched peri-carcinomatous tissues of CRC patients, as well as tissues from AOM/DSS treated mice and reverse-transcribed into cDNA. Then the gene expression levels of STING signaling pathway-associated molecules were determined by quantitative PCR. The data from the same patient were connected by straight lines. The data of the expression of STING signaling pathway-associated molecules were retrieved from the TCGA microarray database in Oncomine® platform. Expression of *STING* **(A, B, C)**, *IFI16* **(D, E, F)**, *DAI* **(G,H, I)** and *DDX41* **(J, K, L)** in human CRC tissues, tissues from AOM/DSS treated mice, and TCGA database. Control: matched peri-carcinomatous tissues, n=44 for clinical samples, data represent two independent experiments; n=7 for PBS-treated mice and n=5 for AOM/DSS- treated mice; n=22 for health normal controls and n=215 for CRC group. *p < 0.05, **p < 0.01, ***p < 0.001; NS: Not significantly different.

**Figure 4:**
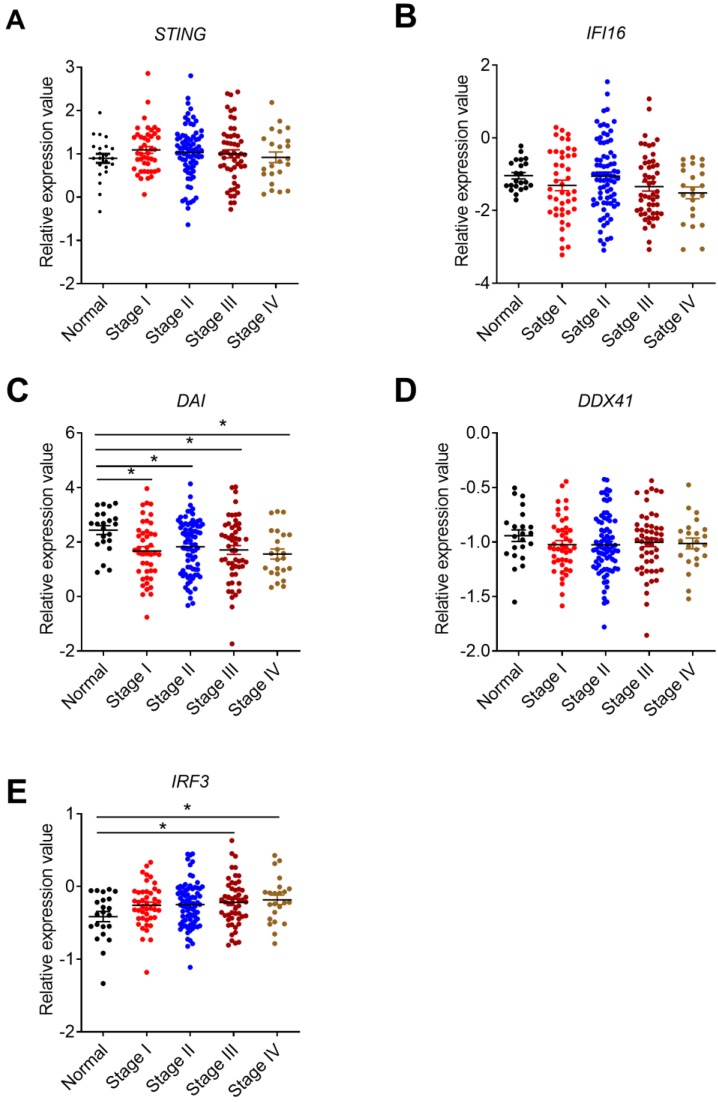
** Correlation between STING signaling-associated molecules expression and cancer stages.** The expression data of STING signaling pathway-associated molecules were retrieved from the TCGA microarray database in Oncomine® platform and were grouped by stages for further analysis. Correlation between *STING* **(A)**, *IFI16* **(B)**, *DAI***(C)**, *DDX41* **(D)**, *IRF3* **(E)**, and cancer stages were shown. n=22 for health control, 44 for stage I, 78 for stage II, 52 for stage III, and 23 for stage IV. Data were expressed as mean ± SEM, Log2 median-centered ratio expression. *p < 0.05.

STING is crucial for the production of type I IFNs and activation of CD8^+^ T cells 38, 39.On one hand, STING can be activated by cyclic-dinucleotides (CDNs) that is secreted by bacteria or synthesized by cyclic GMP-AMP synthase (cGAS) 40 . On the other hand, STING is also the adaptor for some other DNA sensors include DDX41, IFI16, and DAI 27, 22. A previous study indicated that *Sting^-/-^* mice were more susceptible to AOM/DSS-induced CRC 23. In addition, functional defects and variant in STING signaling were frequently observed in human CRC cell lines 41. Our study found that the expression of *STING* displayed no significant change in clinical CRC samples, samples from AOM/DSS treated mice, or TCGA database, and in different stages of CRC (**Figure 3A, B, C, Figure 4A**). These results were different from another recent study, which showed that STING expression was significantly lower in Chinese CRC patients and there were a dysregulation of the cGAS/IFI16-STING-TBK1-IFNβ pathway 42.

Interestingly, the expression level of *cGAS* was undetectable in our CRC samples (Data not shown), which may be partially associated with the methylation of cGAS in CRC cells 41. Furthermore, the methylation of cGAS in some of our CRC samples might also affect the STING expression, thus leading to the discrepancy between our study and previous studies.

Although IFI16 belongs to the member of ALRs, it mainly functions via STING signaling pathway 22. IFI16 plays an important role in virus restriction, however, its role in tumor setting is not well studied. No significant expression difference of *IFI16* were observed in our clinical CRC samples** (Figure 3D)**. In addition, we examined the expression levels of p204, a homolog to human IFI16, in colon tissues of 14 days after AOM/DSS treatment in mice. The results of p204 also showed no significant expression changes in our mouse samples (**Figure 3E**). But its expression levels decreased in TCGA database (**Figure 3F**). However, no correlation between *IFI16* expression level and cancer stages was identified (**Figure 4B**). The role of DAI in tumor setting, especially in CRC, is also unclear. We found that *DAI* expression decreased in the early stage of tissues from AOM/DSS treated mice and TCGA database (**Figure 3H, I)**, whereas no significant change was observed in our clinical samples (**Figure 3G**). Furthermore, *DAI* expression levels were associated with cancer stages (**Figure 4C**).

As a member of helicase family, DDX41 detects dsDNA and signals via STING to mediate the production of Type I IFNs. However, the function of DDX41 is much more complicated and was also reported to participate in RNA metabolism. A recent study found that DDX41 mutation was frequently observed in human hematological malignancy 27. However, the role of DDX41 in solid tumor is still unknown. Our results showed that *DDX41* expression in human CRC was much higher than the control groups (**Figure 3J**) but the association with tumor stages was not identified (**Figure 3H**). DDX41 expression levels had no significant changes in TCGA database (**Figure 4D**). In addition, *Ddx41* expression decreased in AOM/DSS treated mice (**Figure 3K**). The discrepancy between the mouse model and human CRC could be possibly due to the time point difference.

Given that STING-TBK1-IRF3 signaling pathway is important for the production of type I IFNs and CD8^+^ T cell-mediated anti-tumor response, the expression of *IRF3* in CRC was also analyzed in TCGA database. We found that the mRNA level of *IRF3* increased in CRC tissues and was associated with tumor stages (**Figure 4E**).

### The expression profile of helicases family members in CRC

Other DNA sensors in helicase family included DHX9, DHX36, and DDX60. A study suggested that they are indispensable for type I IFN induction and host defense against multiple viral infection 43. In addition, previous studies have shown that DHX9 plays an important role in maintaining gene stability and promotes the survival of tumor cells 44, 45, 46. Recent proteomic studies also suggest elevated DHX9 expression levels in CRC, whereas DHX36 silence reduced CRC invasiveness and metastasis 26, 47. These studies indicate that the helicase family may be also involved in the development of CRC. However, the signaling pathways for these molecules are still largely unknown.

**Figure 5:**
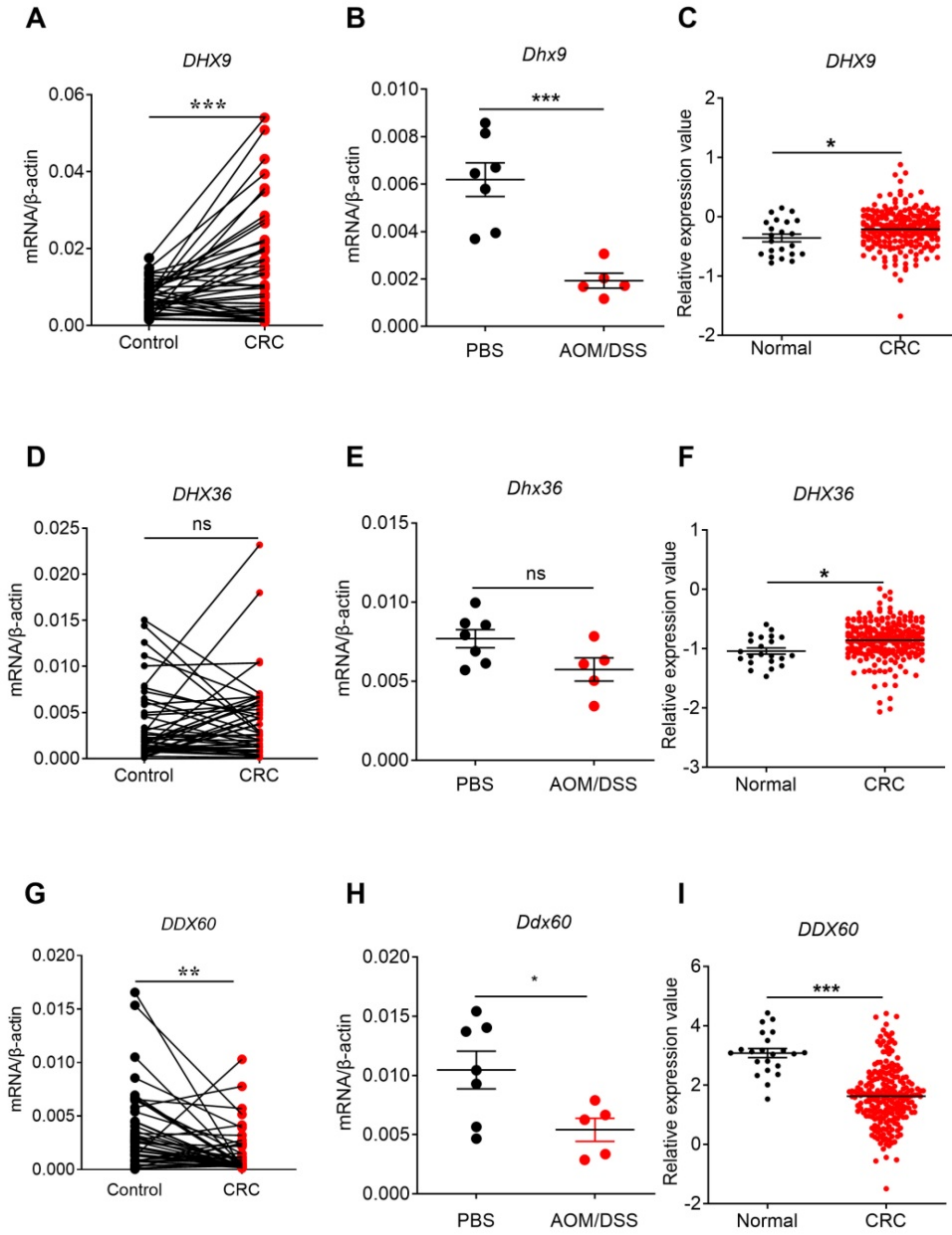
** The expression profile of helicases in human and mouse CRC tissues and TCGA database.** RNA was extracted from cancer and matched peri-carcinomatous tissues of CRC patients, as well as tissues from AOM/DSS treated mice, and reverse-transcribed into cDNA. Then the gene expression levels of helicases were determined by quantitative PCR. The data from the same patient were connected by straight lines. The data of the expression of helicases were retrieved from the TCGA microarray database in Oncomine® platform. Expression of *DHX9* **(A, B, C)**, *DHX36* **(D, E, F)**, and *DDX60* **(G, H, I)**, in human CRC tissues, tissues from AOM/DSS treated mice, and TCGA database. Control: matched peri-carcinomatous tissues, n=44 for clinical samples, data represent two independent experiments; n=7 for PBS-treated mice and n=5 for AOM/DSS- treated mice. *p < 0.05, **p < 0.01, ***p < 0.001; NS: Not significantly different.

**Figure 6:**
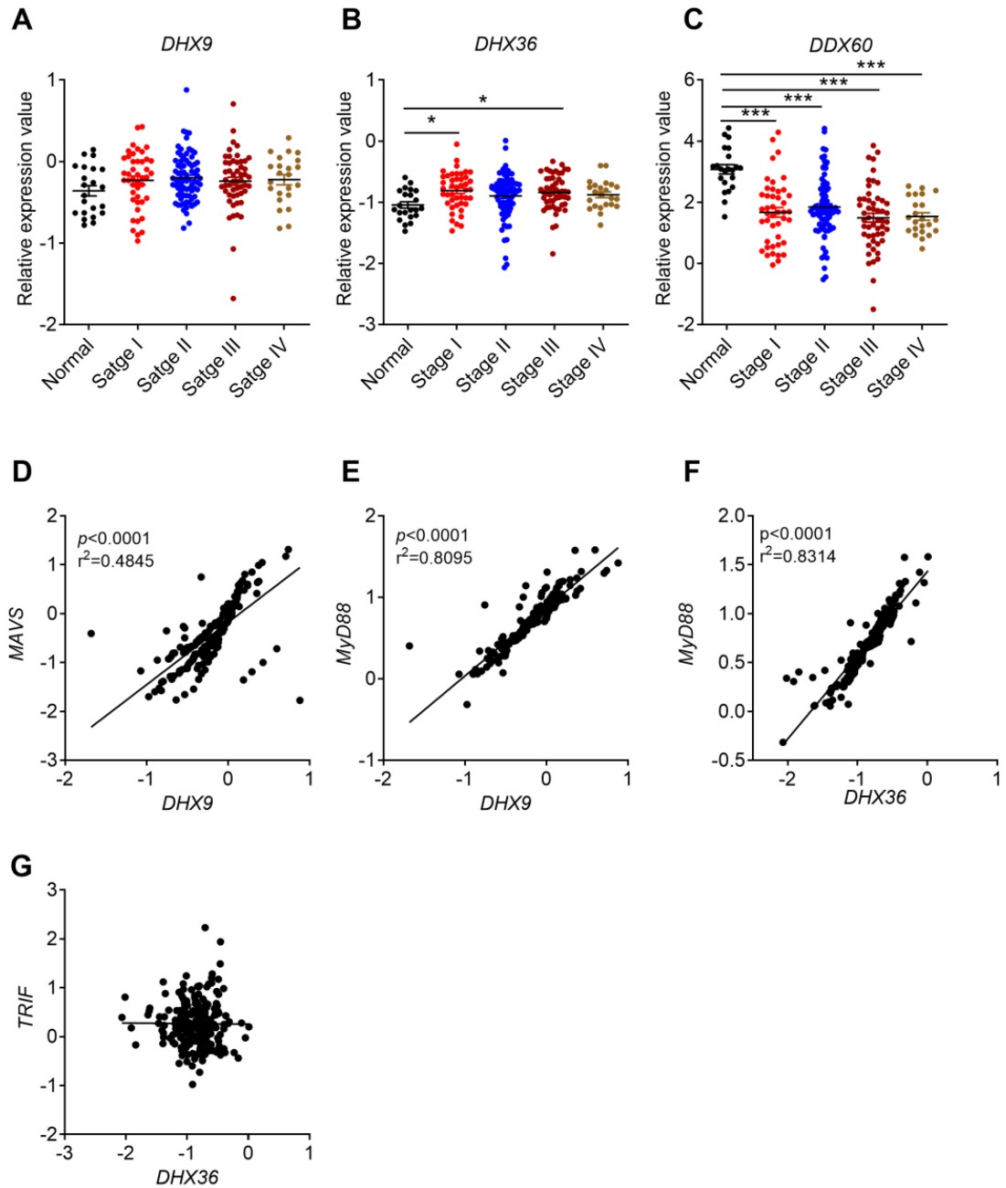
** Correlation between helicases expression and cancer stages, and between their expression and MyD88/MAVS/TRIF expression**. The expression data of helicases were retrieved from the TCGA microarray database in Oncomine® platform, and were grouped by stages or patient IDs for further analysis. **(A-C)** Correlation between *DHX9*, *DHX36*, and *DDX60* and cancer stages were shown. **(D-G)** Expression correlation between *DHX9* and *MAVS/MyD88, DHX36* and *MyD88/TRIF*. n=22 for health normal control, 44 for stage I, 78 for stage II, 52 for stage III, and 23 for stage IV. Data were expressed as mean ± SEM, Log2 median-centered ratio expression. *p < 0.05; ***p < 0.001.

Our present study found that the expression of *DHX9* increased in clinical CRC samples and/or TCGA database (**Figure 5A, C**). The expression of *DHX36* in CRC was not different in our clinical samples or AOM/DSS-treated samples, but increased in ATCG database and was associated with cancer stages (**Figure 5D, E, F and Figure 6B**). Notably, a recent study found that inhibition of DHX9 promoted death of tumor cells but did not affect the function of normal cells at organ level, indicating that DHX9 may have potential to serve as a safe and effective target for CRC therapy 46. Interestingly, the expression of *Dhx9* was decreased in AOM/DSS-treated mice (**Figure 5B**). The differences between the mouse model and human CRC might be due to the time point difference.

Clinical CRC database indicated that DHX9 and DHX36 were upregulated in CRC tissues. However, the expression of *DDX60* decreased in clinical CRC, AOM/DSS treated mouse tissues, and TCGA database (**Figure 5G, H, I**). In addition, the expression of *DDX60* in CRC was associated with cancer stages (**Figure 6C**). These results consistently indicated that DDX60 were downregulated in the development of CRC.

A previous study showed that DHX9 can recruit MAVS and MyD88 after recognizing dsRNA and CpG modified ssDNA, respectively 48. Therefore, we analyzed the correlation between DHX9 and MAVS or MyD88 in CRC. We found that *DHX9* and *MVAS or MyD88* had a positive correlation (**Figure 6D, E**). On the other hand, DHX36 was reported to interact with DDX1 and DDX21 to form a complex that further signals via TRIF or MyD88 49. Our analysis result identified positive correlation between *DHX36* and *MyD88* but not *TRIF* (**Figure 6F, G**).

### The expression profile of TLR9 signaling molecules in CRC

**Figure 7:**
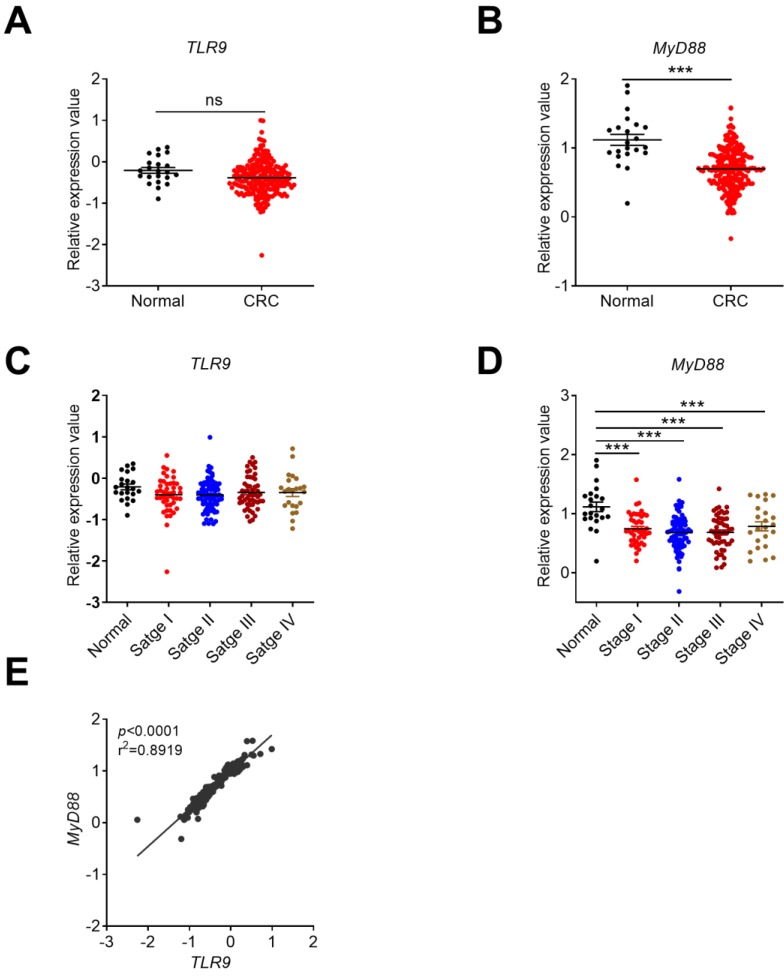
** The expression profile of *TLR9* in CRC tissues and its correlation with tumor stages or* MyD88.*** The expression data of *TLR9* were retrieved from the TCGA microarray database in Oncomine® platform and were grouped by stages or patient IDs for further analysis. Expression of *TLR9* **(A)** and *MyD88* **(B)** in TCGA database. Correlation analysis of *TLR9***(C)** and *MyD88* **(D)** in different stages of CRC and health control. **(E)** Expression correlation between* TLR9* and *MyD88*. n=22 for health normal control, 44 for stage I, 78 for stage II, 52 for stage III, and 23 for stage IV. Data were expressed as mean±SEM, Log2 median-centered ratio expression. ***p < 0.001; NS: Not significantly different.

Our previous study showed that the expression of TLR9 in human CRC and AOM/DSS-induced early stage of CRC tissues significantly decreased 35. Subsequently, we further analyzed the expression profile of *TLR9* in clinical CRC samples, AOM/DSS-induced mice, and TCGA database and its adaptor *MyD88* in TCGA database in Oncomine platform. No significant change of *TLR9* was observed in TCGA database (**Figure 7A**). No correlation exists between *TLR9* expression and cancer stages in Oncomine platform (**Figure 7C**). The expression of adaptor *MyD88* was significant decreased in CRC group (**Figure 7B**) and associated with cancer stages (**Figure 7D**). Further analysis showed that a positive correlation existed in *TLR9* and *MyD88* in CRC tissues (**Figure 7E**). The overall expression profiles of DNA sensors were summarized (**Table 1**).

### The protein levels of DNA sensors in human CRC tissues

**Figure 8:**
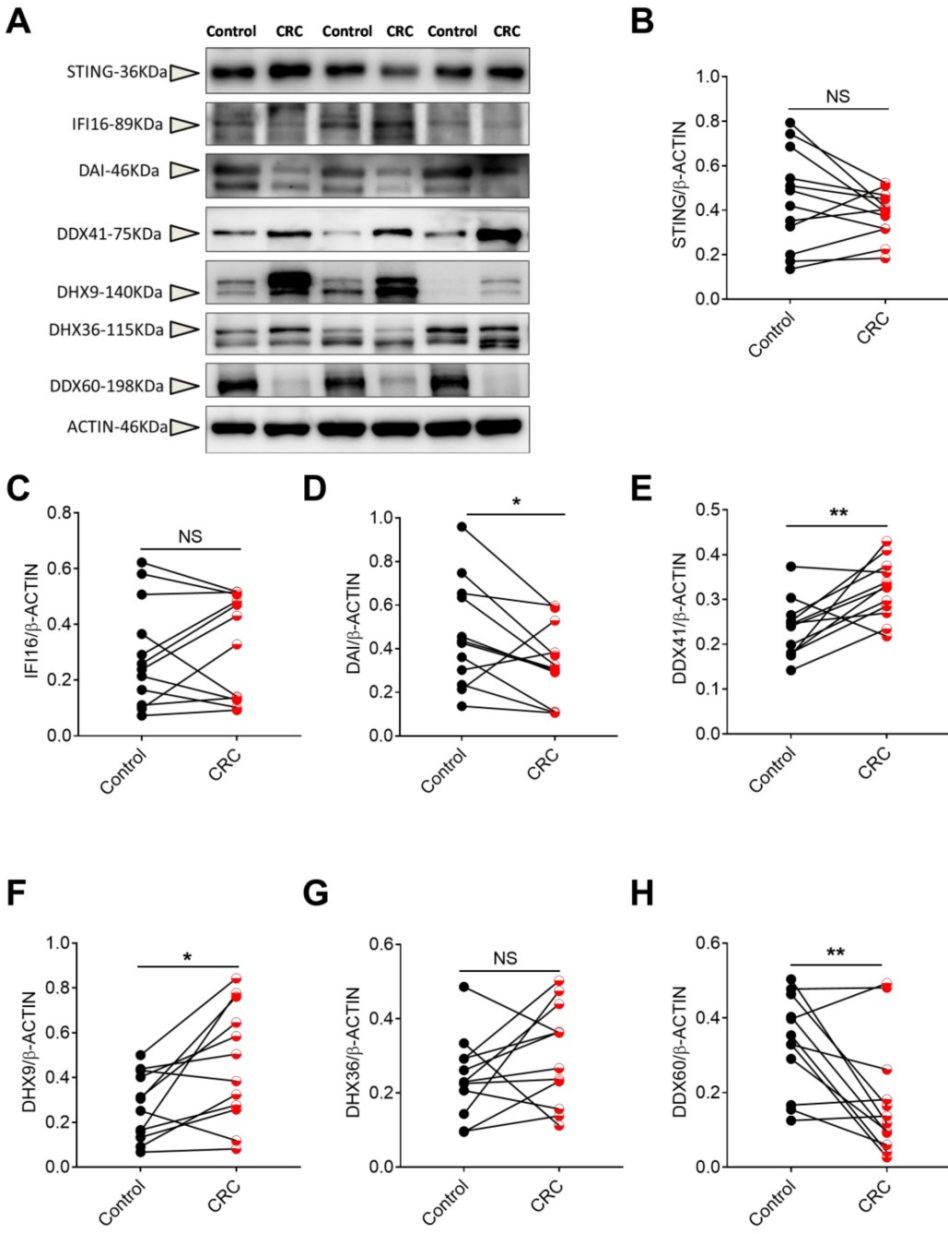
** The protein levels of DNA sensors in human CRC tissues.** Protein was extracted from cancer and matched peri-carcinomatous tissues of CRC patients, and then the levels of DNA sensors including STING, IFI16, DAI, DDX41, DHX9, DHX36 and DDX60 were determined by Western-blot. **(A)** The representative Western-blot results. The triangles point to the specific bands of the molecules. Densitometric analysis of band intensity of STING **(B)**, IFI16**(C)**, DAI**(D)**, DDX41**(E)**, DHX9**(F)**, DHX36**(G)** and DDX60**(H)** was shown. Control: matched peri-carcinomatous tissues, CRC: colorectal cancer tissues, n=12. *p < 0.05; **p < 0.01; NS: Not significantly different.

To confirm the gene expression profile, we also tested the protein level of DNA sensors including STING, IFI16, DAI, DDX41, DHX9, DHX36 and DDX60 in CRC and the matched pericarcinomatous tissues using Western-blot analysis. We found that the protein changes of these DNA receptors in CRC were consistent with the gene expression patterns (**Figure 8**).

## Discussion

Aberrant inflammatory response is an important risk factor of tumor formation, as well as an important feature after tumor formation 50. In the early stage of tumor formation, inflammatory environment facilitates the activation of various signaling pathways, and the differentiation from normal cell into hyperplasia toward tumor 50. In the late stages of tumor formation, tumor cells require a variety of positive or negative regulatory mechanisms to escape immune surveillance 51. The dysregulation of the immune system compromises the effective host barrier against the formation and development of tumors.

DNA sensors are one of the major sub-family of innate immune receptors that recognize DNA. They are mainly expressed in the cytoplasm of innate immune cells and can be also expressed in some other cells, such as epithelial cells. The activation of these receptors can lead to the elimination of invading pathogenic microorganisms through recognizing exogenous DNA. Subsequent studies have shown that the DNA sensors can also respond to abnormal accumulated endogenous DNA in cytoplasm and is involved in the development of inflammatory diseases and autoimmune diseases 52. Notably, in recent years, more and more studies have shown that DNA sensors also participate in the development of tumors, although their specific functions and mechanisms are still unknown.

Recent studies suggest that DNA sensors such as AIM2, TLR9, DHX9, DHX36 and adaptor STING may have roles in CRC development, and the other DNA sensors such as DDX41, DDX60, IFI16, and DAI participate in the development of other types of cancers. Here, we firstly highlighted the notion that many DNA sensors may have a regulatory role in the occurrence and development of CRC. However, there is still a lack of systematic understanding on the expression profile, underlying mechanisms and clinical significance of DNA sensors in CRC. Our present study represents a comprehensive analysis of DNA sensor expression in CRC and its relationship with the tumor stages by combining with the clinical CRC samples, mouse CRC samples, and Oncomine platform.

We found that DNA sensors and the downstream molecules, including *AIM2*, *DDX60*, *TLR9*, *ASC*, *MyD88*, and *IL-18* were down-regulated in CRC. The helicase family members, including *DDX41*, *DHX9*, and *DHX36*, were up-regulated in CRC tissues in comparison with controls. IFI16 and *STING* were the exception in that they showed no significant change in CRC. Our results of STING and IFI16 were different from some previous studies. Genetic and epigenetic difference may exist by regions, racial or the quality of sample. Further analysis showed that the expression of *AIM2*, *DDX41, DHX9, DAI*, and *DDX60* showed significant differences between controls and CRC samples. Among these DNA sensors, AIM2, DAI, and DDX60 showed consistent change in our clinical CRC samples and TCGA database, and were worthy of further investigation on their role in CRC development and clinical application.

In summary, our present work studied the expression profile of DNA sensors in CRC, which broaden our horizons about the function of DNA sensors beyond immunity against viral infection. Notably, although some agonists of DNA sensors (e.g. TLR9 and STING) have been applied in clinical or pre-clinical studies and achieved some encouraging outcomes, their effects in different tumors or different stages are largely undefined. Therefore, further understanding on the expression and the role of DNA sensors in different cell types in TME is necessary for rational design of DNA sensor agonists in clinical trials. On the other hand, we noticed that although* DHX9*, were up-regulated in CRC, they decreased in early stage of AOM/DSS treated mouse. Therefore, the expression of DNA sensors may vary along with diseases progression, and further investigations on their expression patterns in middle and late stages of CRC formation may help to understand the full DNA sensors expression patterns.

## Supplementary Material

Supplementary tables.

## Figures and Tables

**Table 1:** The summary of expression profiles of DNA sensors and downstream molecules and their correlations with cancer stages in CRC

Genes		Human CRC tissues	TCGA database	Mouse AOM/DSS treatment	Associated with Cancer stages
**ALRs**	AIM2	Downregulated	Downregulated	Downregulated	No
	IFI16	NS	Downregulated	NS	No
	TLR9	Downregulated	NS	Downregulated	No
	STING	NS	NS	NS	No
	DAI	Downregulated	Downregulated	Downregulated	Yes
**DDXs** **DHXs**	DDX41	**Upregulated**	NS	Downregulated	No
	DHX9	**Upregulated**	**Upregulated**	Downregulated	No
	DHX36	NS	**Upregulated**	NS	Yes
	DDX60	Downregulated	Downregulated	Downregulated	Yes
	MyD88	/	Downregulated	/	Yes
	IRF3	/	Upregulated	/	Yes
	ASC	/	Downregulated	/	Yes
	IL-18	/	Downregulated	/	Yes

(Note: NS, not statistically significant)
