# Supplementary material for: DNA sensors, crucial receptors to resist pathogens, are deregulated in colorectal cancer and associated with initiation and progression of the disease
Source: J Cancer. 2020 Jan 1;11(4):893–905. doi: 10.7150/jca.34188 (PMC6959017; doi:10.7150/jca.34188)
Supplement: Supplementary file 1 — Supplementary tables. [file jcav11p0893s1.pdf]

Supplemental Table 1. The clinical pathological features of CRC samples

Gender	Number
Male	20（45.5%）
Female	24（54.5%）
**Age (years)**	
<50	10(22.7%)
≥50	34(77.3%)
**Location**	
Colon	23(52.3%)
Rectal	21(47.7%)
**Clinical Stages**	
II	23(52.3%)
III	14(31.8%)
IV	7(15.9%)
**Grades**	
1	13(29.5%)
2	24(54.5%)
3	7(15.9%)
**Pathologic Type**	
Adenocarcinoma	36(81.8%)
Mucinous Adenocarcinoma	8(18.2%)

Supplemental Table 2. The Primer sequences of DNA sensors (human)

Genes (Human)	Sequences (5'-3')
**AIM2**	(F): TGG CAA AAC GTC TTC AGG AGG
	(R): AGC TTG ACT TAG TGG CTT TGG
**STING**	(F): CCA GAG CAC ACT CTC CGG TA
	(R): CGC ATT TGG GAG GGA GTA GTA
**DHX9**	(F): CGA ACC ATC TCA GCG ACA AAA
	(R): TGA GGT CCA TGC TTA TTT GCT C
**DDX41**	(F): GTG CCC TAT GTG CCG TTA C
	(R): GGC TGA CGT TGG ACT GAG G
**DDX60**	(F): CAG CTC CAA TGA AAT GGT GCC
	(R): CTC AGG GGT TTA TGA GAA TGC C
**TLR9**	(F): CTG CCT TCC TAC CCT GTG AG
	(R): GGA TGC GGT TGG AGG ACA A
**DAI**	(F): AAC ATG CAG CTA CAA TTC CAG A
	(R): AGT CTC GGT TCA CAT CTT TGC
**IFI16**	(F): AGA CTG AAG ACT GAA CCT GAA GA
	(R): GAA CCC ATT GCG GCA AAC ATA
**DHX36**	(F): GGG TCA TGG AGG TAA CCG AG
	(R): CTC TCC GCT TCC TTG TTC TTC
**β-actin**	(F): CTC CTT AAT GTC ACG CAC GAT
	(R):CAT GTA CGT TGC TAT CCA GGC

Supplemental Table 3. The Primer sequences of DNA sensors (mouse)

Genes (Mouse)	Sequences (5'-3')
**AIM2**	(F): GTC ACC AGT TCC TCA GTT GTG
	(R): CAC CTC CAT TGT CCC TGT TTT AT
**STING**	(F): GGT CAC CGC TCC AAA TAT GTA G
	(R): CAG TAG TCC AAG TTC GTG CGA
**DHX9**	(F): TAT CGT GGT ATG GGC AAT TCC A
	(R): GTG TCG CTA AGT ATG GGT GGG
**DDX41**	(F): AGT CCG CCA AGG AAA AGC AA
	(R): CTC AGA CAT GCT CAG GAC ATA AC
**DDX60**	(F): TTC CAC TGC CCA AAA TAG GAA AA
	(R): GCC AGC AAC ATG AGT CTT AGG AT
**TLR9**	(F): ATG GTT CTC CGT CGA AGG ACT
	(R): GAG GCT TCA GCT CAC AGG G
**DAI**	(F): AAG AGT CCC CTG CGA TTA TTT G
	(R): TCT GGA TGG CGT TTG AAT TGG
**IFI204**	(F): AAA GAG ACA ACC AAG AGC AAT ACA CC
	(R): GAA TGT TAG ATG AAG CCG AAG ATG AG
**DHX36**	(F): ATG GAT GAA CGT CGA GAA GAG C
	(R): ATA CCC ATG ATC CTC AGG AGC
**β-actin**	(F): CAG CTT CTT TGC AGC TCC TT
	(R): CAC GAT GGA GGG GAA TAC AG
